# A giant liposarcoma originating from peripancreatic fat tissue with identification using 3D-CT angiography: a case report

**DOI:** 10.1186/s40792-023-01797-3

**Published:** 2024-01-08

**Authors:** Kiyoshi Narita, Hiroshi Kaneko, Fumiya Hasegawa, Nozomi Akao, Tomoki Kusafuka, Ryosuke Desaki, Masaomi Ogura, Takashi Hamada, Kana Asakawa, Tetsuya Murata

**Affiliations:** 1 Department of Surgery, JA Suzuka General Hospital, 1275-53 Yasuzukacho Yamanohana, Suzuka, Mie 513-8630 Japan; 2 Department of Pathology, JA Suzuka General Hospital, 1275-53 Yasuzukacho Yamanohana, Suzuka, Mie 513-8630 Japan

**Keywords:** Liposarcoma, Peripancreatic fat tissue, 3D-CT angiography, Dorsal pancreatic artery, Giant tumor

## Abstract

**Background:**

Liposarcoma originating from peripancreatic fat tissue is extremely rare. This case report presents a surgical case of a giant liposarcoma originating from peripancreatic fat tissue with origin identification using 3-Dimensional Computed Tomography Angiography (3D-CTA).

**Case presentation:**

A 59-year-old female was referred to our hospital with a giant abdominal tumor. Computed tomography revealed a 34 cm tumor composed of fatty tissue, exerting pressure on the posterior aspect of the pancreas. Suspecting liposarcoma, we planned for surgery. At first, the tumor appeared to be intra-abdominal tumor, based on the identification of the tumor’s feeding artery as a branch of the dorsal pancreatic artery using 3D-CTA, we concluded that the liposarcoma originated from the peripancreatic fat tissue and situated in the retroperitoneum. During surgery, we observed a well-capsulated, elastic, yellowish mass without infiltration into surrounding tissues. We carefully dissected the tumor from the greater omentum and transverse mesocolon while preserving the tumor capsule. We ligated the feeding artery at the border with the pancreatic parenchyma and successfully completed the excision of the tumor. The resected specimen weighted 2620 g and was pathologically diagnosed as a well-differentiated liposarcoma. There was no injury to the tumor’s capsule, and the surgical margins were negative.

**Conclusions:**

In this report, we present an extremely rare case of a liposarcoma originating in the peripancreatic fat tissue. The use of 3D-CTA was instrumental in identifying the primary site of this giant tumor, enabling us to guide the surgery and achieve complete resection successfully.

## Background

The first-line treatment for liposarcoma is surgery; performing en bloc resection with the capsule is of utmost importance. We experienced a rare case of a giant liposarcoma originating from the peripancreatic fat tissue. In this instance, the preoperative identification of the tumor’s feeding artery using 3-Dimensional Computed Tomography Angiography (3D-CTA) played a crucial role in determining its origin and contributed to the successful complete resection. We present this significant in this report.

## Case presentation

**Fig. 1:**
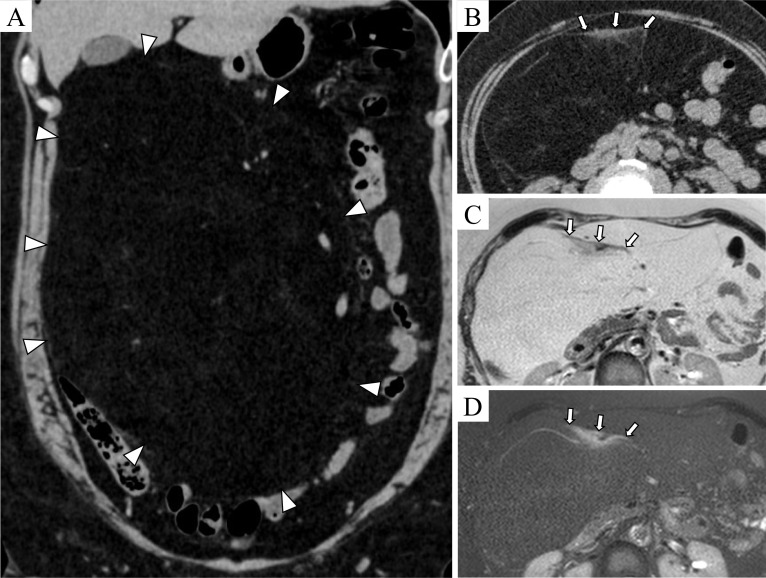
Abdominal CT and MRI. **A**, **B** Abdominal contrast-enhanced CT. **C** Abdominal plain MRI T2-weighted image. **D** Abdominal plain MRI fat-suppressed T2-weighted image. A giant tumor measuring 34 cm predominantly composed of fat tissue was observed in the abdomen (white triangles). A high density area of the ventral side of the tumor was detected on CT (white arrows), and the region showed equivalent-signal intensity area to renal parenchyma on MRI T2-weighted image and not suppressed on fat-suppressed images (white arrows)

A 59-year-old female with a history of chest X-ray examination revealing bilateral lung ground-glass appearance 4 years ago was undergoing regular chest computed tomography (CT) for follow-up observation. On the recent CT, a giant abdominal tumor was identified, and she referred to our hospital. Her height was 158 cm, and body weight was 66 kg. The abdominal examination revealed a flat and soft abdomen, with no palpable mass detected on the surface. Laboratory findings showed no significant abnormalities. An abdominal contrast-enhanced CT revealed a 34 cm tumor with a similar density to subcutaneous fat (Fig. 1). A high density area was observed on the ventral side of the tumor. Abdominal plain magnetic response imaging (MRI) revealed that the tumor was suppressed on the fat-suppressed images, indicating a predominance of fatty components. The area that showed high density on CT exhibited equivalent-signal intensity area to renal parenchyma on T2-weighted images, and not suppressed on fat-suppressed images. The upper and lower gastrointestinal endoscopy revealed no abnormalities without hiatal hernia.

**Fig. 2:**
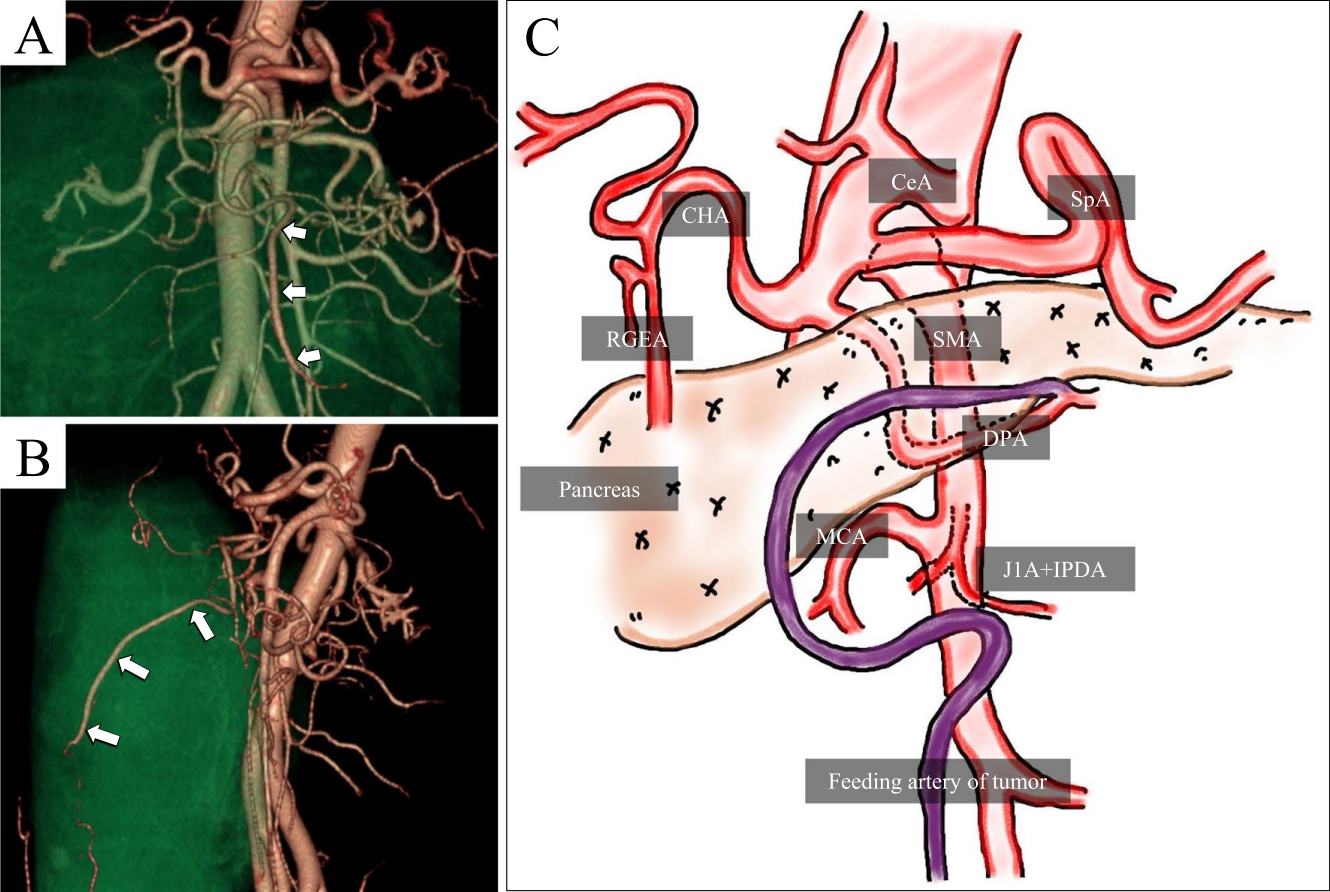
3D-CT angiography. **A** Coronal image, **B** Sagittal image, **C** Schema. The tumor’s feeding artery was identified as a branch of the DPA. *CeA* celiac artery, *SpA* splenic artery, *CHA* common hepatic artery, *RGEA* right gastroepiploic artery, *SMA* superior mesenteric artery, *DPA* dorsal pancreatic artery, *MCA* middle colic artery, *J1A* first jejunal artery, *IPDA* inferior pancreaticoduodenal artery

**Fig. 3:**
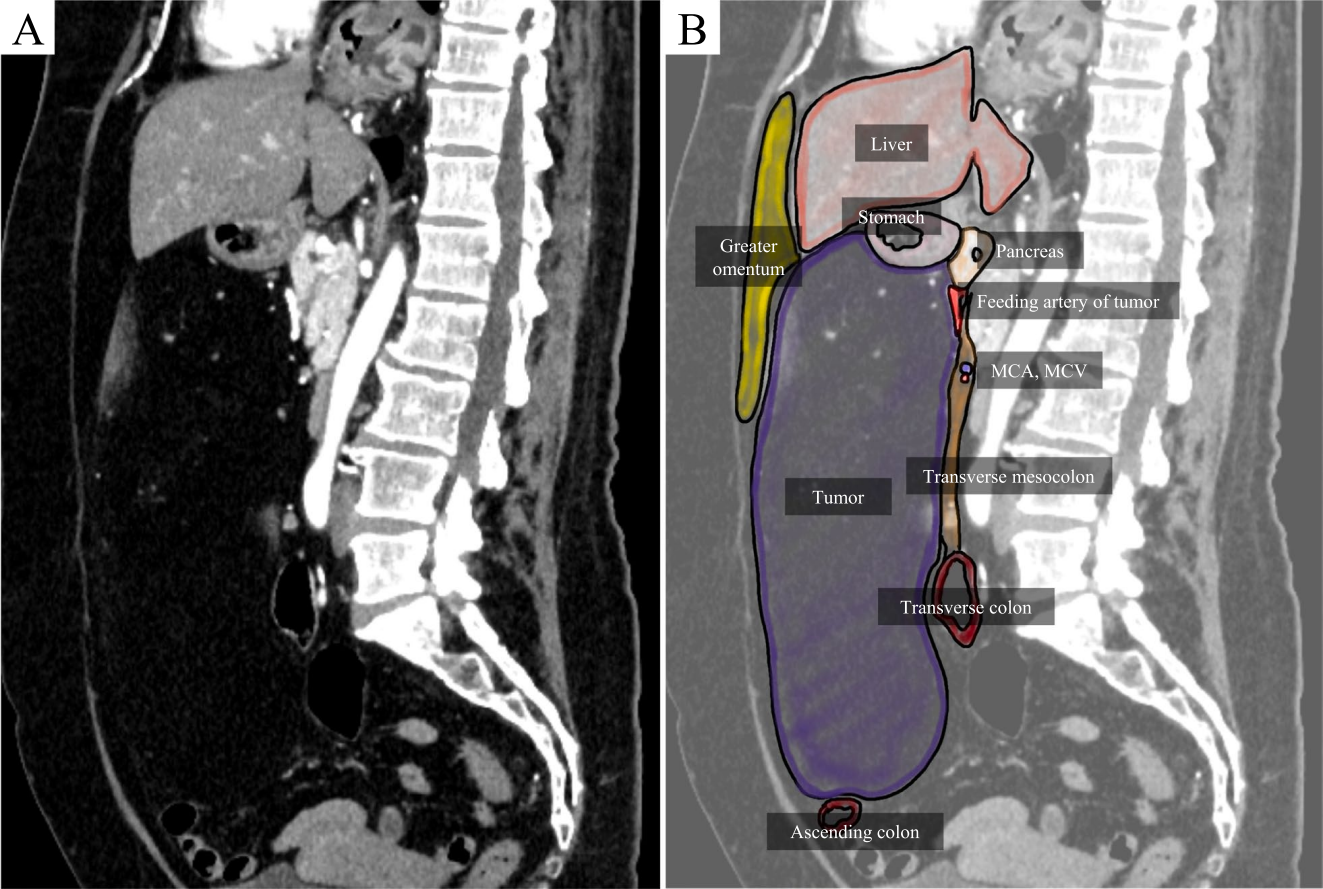
Preoperative CT and schema. **A** Abdominal contrast-enhanced CT sagittal image, **B** schema. The tumor was located on the ventral side of pancreas. Transverse colon and transverse mesocolon were displaced dorsally and stomach and greater omentum were compressed ventrally by the tumor. Based on its localization and vascular supply, the tumor was considered to originate from the peripancreatic fat tissue and situated in the retroperitoneum

The tumor was suspected of liposarcoma and decided to proceed with surgery. The tumor appeared to be composed of homogeneous fatty tissue, and there was no clear evidence of infiltration into the surrounding organs on imaging. Therefore, a well-differentiated liposarcoma was the most likely diagnosis, but the areas showing high density on CT and not suppressed on MRI fat-suppression images were considered to potentially indicate dedifferentiation. Artery running through the tumor was recognized, and 3D-CTA showed that the tumor was supplied by a branch of the dorsal pancreatic artery (DPA) (Fig. 2). The branches of the right gastroepiploic artery (RGEA) and superior mesenteric artery (SMA), such as the middle colic artery (MCA), were observed to be compressed and displaced by the tumor, and they were not supplying blood to the tumor. Regarding the origin of the tumor, at first, given the appearance of the tumor occupying the intraperitoneal space, we thought it originated from an intra-abdominal organ. However, considering that the tumor’s feeding artery was branches of the DPA, we concluded that the tumor originated retroperitoneally. Upon reevaluating the CT images, we suspected that the tumor arose from the peripancreatic fat tissue, exerting pressure on the stomach and greater omentum ventrally and displacing the transverse colon and transverse mesocolon dorsally (Fig. 3). In addition, the drainage vein was flowing into the middle colic vein (MCV).

**Fig. 4:**
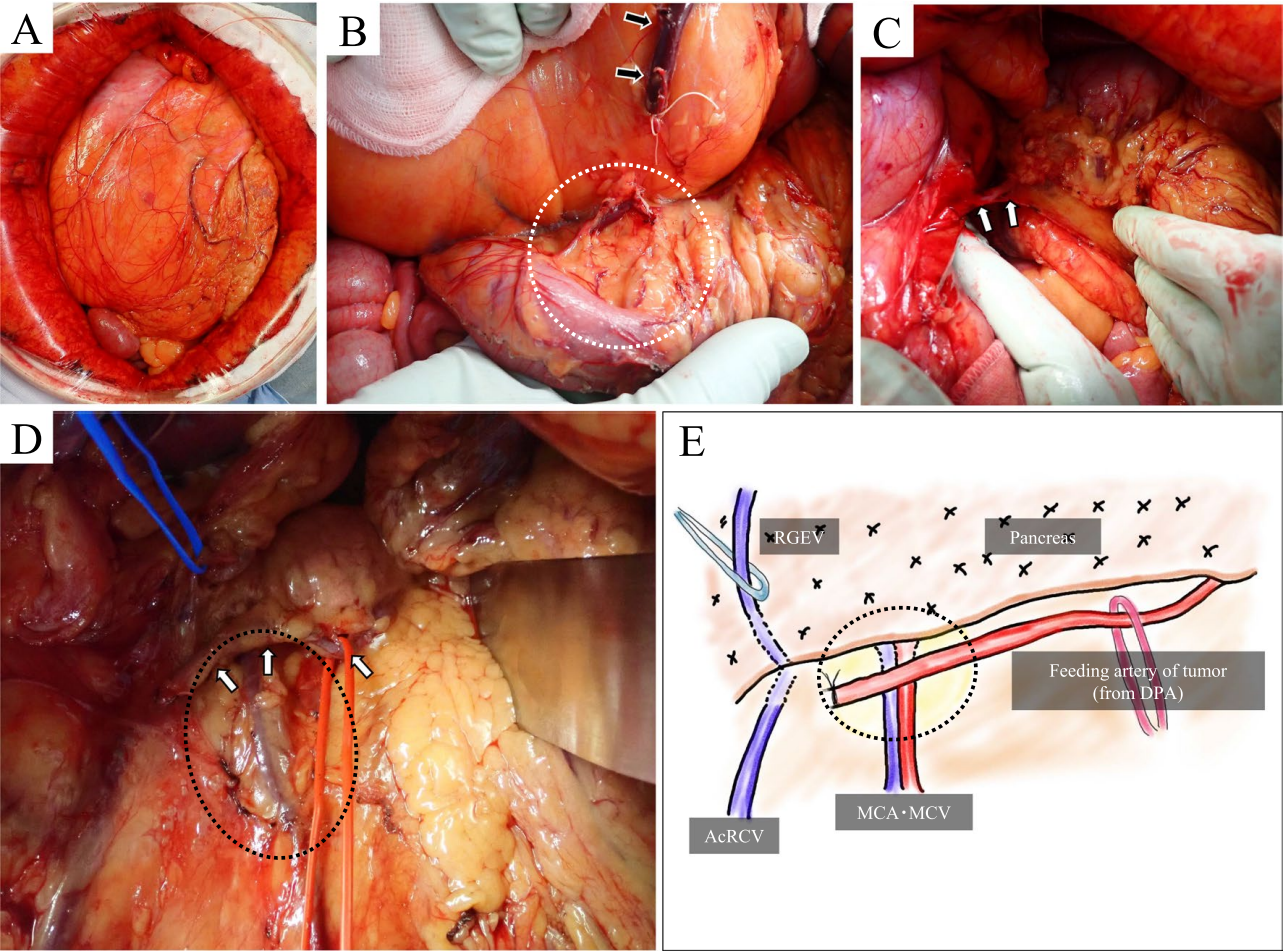
Intraoperative photographs. **A** The tumor was yellow and approximately size of an adult’s head. **B** We dissected of the transverse mesocolon (dotted white circle) and ligated drainage vein (black arrows) at the site where it flows into the transverse mesocolon. **C** We identified the feeding artery at the lower border of the pancreas (white arrows). **D**, **E** (Schema): after tumor resection, we clarified the relationship between the feeding artery (white arrows) and other vessels. We also removed the portion of the pancreatic capsule and the transverse mesocolon attached to the tumor, which were considered to be the origin of the tumor (dotted black circle). *RGEV* right gastroepiploic vein, *DPA* dorsal pancreatic artery, *AcRCV* accessary right colic vein, *MCA* middle colic artery, *MCV* middle colic vein

She underwent midline laparotomy from the xiphoid process to the lower abdomen, and the tumor was found to be a soft, yellowish mass, approximately the size of an adult’s head, with an extremely thin but distinct capsule (Fig. 4). It did not invade the abdominal wall or other organs. The greater omentum and the tumor capsule could be easily separated without causing injury, as their color and texture differed from the surrounding tissue. Similarly, during the dissection of the transverse colon and transverse mesocolon, separation at a layer that did not injure the visceral peritoneum or tumor capsule was mostly possible. The vein on the back of the tumor was found to be flowing into the transverse mesocolon, confirming the preoperative assessment of the venous drainage into the MCV. To avoid injuring the tumor capsule, the transverse mesocolon was partially attached to the tumor and dissected. Once the dissection between the greater omentum, transverse colon, and its mesentery, and the tumor was completed, only an artery remained at the lower border of the pancreas. Based on the preoperative 3D-CTA, it was determined that this artery was branch of the DPA suppling as the feeding artery. The point of origin of the feeding artery was the fat tissue at the lower border of the pancreas, which was initially suspected as the primary site of the tumor. Therefore, a portion of the pancreatic capsule and the transverse mesocolon were attached to the tumor, and the feeding artery was dissected without injuring the tumor capsule, leading to successful tumor resection. After the tumor was resected, the right gastroepiploic artery and vein (RGEV), MCA, MCV, accessary right colic vein (AcRCV) were clearly visualized, confirming that the feeding arteries of the tumor were distinct from these vessels. The operation time was 1 h and 47 min, with a blood loss of 30 g.

**Fig. 5:**
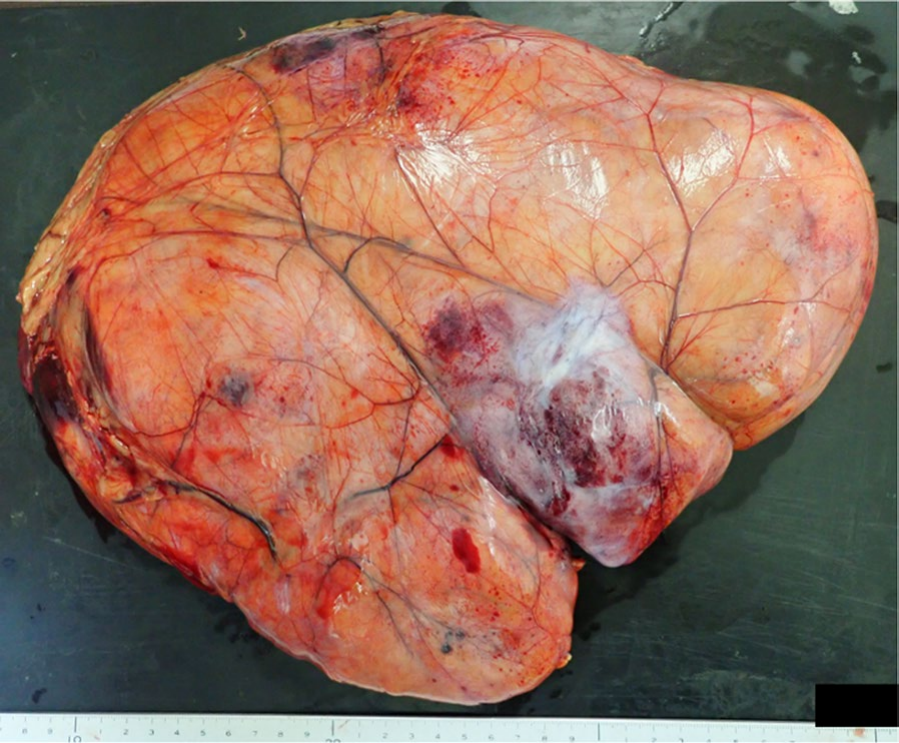
Resected specimen. The size of tumor was 34 × 23 × 7 cm, and its weight was 2590 g. Although the tumor had a thin fibrous capsule, we were able to resect it without injuring the capsule

**Fig. 6:**
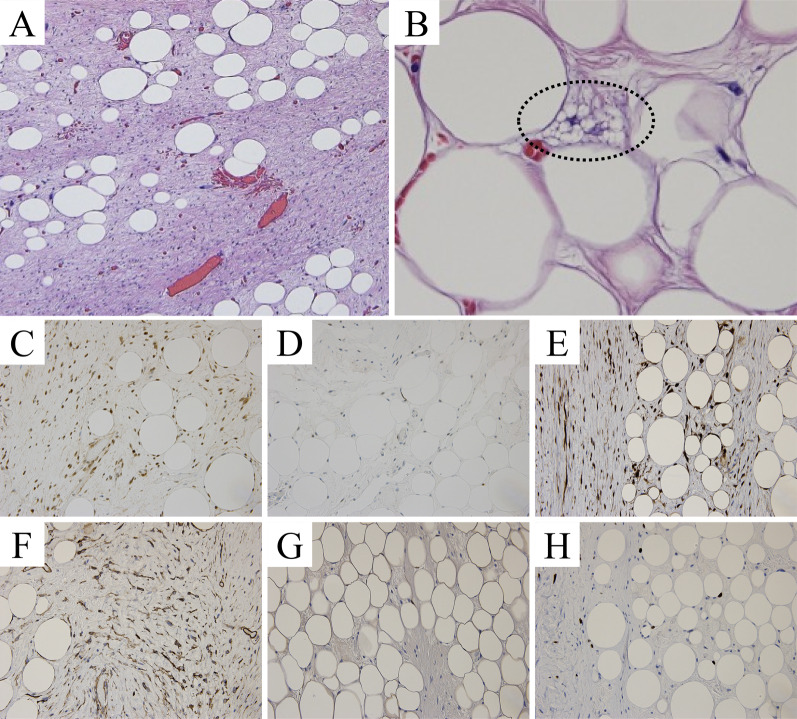
Histopathological and immunohistochemical examinations. **A**, **B**: Hematoxylin and eosin stain (×20). The tumor was mostly composed of slightly enlarged adipocytes of varying sizes, along with atypical stromal cells and lipoblast (dotted black circle). **C** CDK4 positive, **D** MDM2 positive, **E** p16 positive, **F** CD34 weak positive, **G** S-100 weak positive, **H** Ki-67 index of 2%. The tumor was diagnosed as a well-differentiated liposarcoma

The resected specimen measured 34 × 23 × 7 cm, 2620 g (Fig. 5). The tumor had a thin fibrous capsule, and its cut surface was predominantly composed of yellowish fatty tissue, with some areas showing white nodules. Histopathological examination revealed that the tumor was mostly composed of slightly enlarged adipocytes of varying sizes, along with atypical stromal cells and lipoblast (Fig. 6). The white nodules corresponded to the high density areas observed in the CT and showed an increase in fibrous tissue, but no signs of necrosis, nuclear atypia, or mitotic figures were observed, and no dedifferentiation was evident. Immunohistochemical examination revealed positive results for CDK4, MDM2, and p16, weakly positive results for CD34 and S-100, with a Ki-67 index of 2%. Based on these findings, the tumor was diagnosed as a well-differentiated liposarcoma. The tumor was resected without injuring the capsule, and the surgical margins were negative, indicating complete resection.

The postoperative course was uneventful, and she was discharged on the third day after surgery. She did not receive any postoperative therapy. Follow-up evaluations were performed with CT or MRI every 3 months. Two years postoperatively, she remains alive with no evidence of disease recurrence.

## Discussion

Liposarcoma is one of the most common soft tissue sarcomas, accounting for approximately 10% of soft tissue sarcomas [1]. 15% of primary sarcoma arise from retroperitoneum, and 25–35% of liposarcoma consist of soft tissue sarcomas located in retroperitoneal cavity [2]. The early symptoms of retroperitoneal liposarcomas are nonspecific, and the complaints of patients often involve a significant increase in tumor size, leading to direct invasion or compression of adjacent organs [3, 4]. Therefore, retroperitoneal liposarcomas are often diagnosed at advanced stages when tumors develop to large size [5]. In our case, despite the tumor’s enormous size (34 cm), the patient remained asymptomatic, and the diagnosis was incidentally made during CT performed for an unrelated issue. The tumor, occupying the abdominal cavity extensively, initially suggested an origin from intra-abdominal organs. However, it was actually originated from peripancreatic fat tissue and arose in the retroperitoneum. Histologically, liposarcomas are classified into well-differentiated, myxoid, pleomorphic, and round cell types, each with varying prognoses [6]. The 5-year survival rates are closely related to the histological subtypes, with well-differentiated type showing a favorable outcome (85%), compared to myxoid (77%), pleomorphic (18%), and round cell types (21%) [6]. CT is the most common used imaging modalities for the diagnosis and preoperative evaluation [7]. Previous studies have identified certain CT findings suggestive of malignancy, including internal heterogeneity, irregular margins, higher density than normal fat tissue, and enhancement on contrast-enhanced CT [8]. In our case, there were regions with high density on CT and non-suppressed on fat-suppressed MRI, raising suspicion of dedifferentiated liposarcoma. However, the resected specimen confirmed a well-differentiated liposarcoma. The main treatment for non-metastatic retroperitoneum liposarcoma is surgery [9]. It is important to completely resect the tumor with its capsule, and often adjacent organ resection is necessary [7]. The recurrence rate after curative resection is approximately 70%, and this high recurrence rate can be attributed to several reasons, such as the pseudo-capsule formed by tumor cells, the difficulty in ensuring sufficient margins due to the presence of important organs, and the unclear boundary with normal fat tissue [6, 10]. Despite performing extensive resections, there have been reports of local recurrences, suggesting that the recurrence may also be influenced by the malignant potential of the tumor [11]. In our case, with the aid of accurate preoperative localization and well-circumscribed capsule, an appropriate dissection plane was identified. Consequently, there was no need for combined resection of adjacent organs to ensure surgical margin. The histological diagnosis of liposarcoma is considered useful through the detection of protein overexpression due to CDK4 and MDM2 gene amplification [12]. The positivity rate for liposarcoma is approximately 90%, while benign lipomas show a positivity rate of 2–4% [12].

The significance of our report is the identification of the origin of giant abdominal liposarcoma using preoperative 3D-CTA and its application in surgery. Therefore, we conducted a literature review on giant abdominal liposarcomas. 55 cases of primary liposarcoma reported were obtained in PubMed between 2002 and 2023 using the keywords “giant/huge”, “liposarcoma” and “abdomen”, including our case (Table 1) [1–3, 7, 9, 13–59]. The median age was 55 years, with no gender difference observed. The most common origin of liposarcoma was found to be the retroperitoneum in 39 cases (70.6%). The patient’s complaint most frequently reported was abdominal distention, and there were 5 cases (9.1%) where the diagnosis was incidental during unrelated examinations as our case. Regarding surgical procedures, 23 cases (41.8%) underwent tumor resection, while 29 cases (52.7%) required resection involving adjacent organs. Preoperative diagnosis was conducted in 34 cases (61.8%), and the identification of feeding artery was observed in only 3 cases (5.5%). While preoperative site diagnosis was established for cases clearly originating from retroperitoneal fat tissue, such as around the kidney, a considerable number of cases involving retroperitoneal liposarcomas protruding into the abdominal cavity, as our case, were commonly subjected to laparotomy for “abdominal tumor” excision. Intraoperatively, these cases were diagnosed as originating from the retroperitoneum. The median tumor diameter was 32 cm, and the median tumor weight was 9.9 kg. Among the histological subtypes, well-differentiated liposarcoma was the most common, accounting for 24 cases (43.6%), followed by dedifferentiated liposarcoma with 18 cases (32.7%).

**Table 1:** Reported cases of Giant Abdominal Liposarcoma (2002–2023, including our case)

Age, median, year-old	55	(30–55)
Gender		
Male (%)	28	(50.9%)
Female (%)	27	(49.1%)
Origin		
Retroperitoneum	39	(70.9%)
Mesentery	12	(21.8%)
Greater omentum	4	(7.3%)
Patient's complaint *overlapping		
Abdominal distention	26	(47.3%)
Abdominal mass palpation	16	(29.1%)
Abdominal pain	13	(23.6%)
Loss of body weight	10	(18.2%)
Anorexia	6	(10.9%)
None (Incidentally detected during unrelated examination)	5	(9.1%)
Gain of body weight	4	(7.3%)
Others	12	(21.8%)
Operative procedure		
Tumor resection	23	(41.8%)
Tumor resection with other organs	29	(52.7%)
Not mentioned	3	(5.5%)
Preoperative diagnosis of tumor's origin	34	(61.8%)
Preoperative identification of feeding artery	3	(5.5%)
By contrast-enhanced CT	1	(1.8%)
By angiography	1	(1.8%)
By 3D-CT Angiography	1	(1.8%)
Tumor size, median, centimeter	32	(10–66)
Tumor weight, median, kilogram	9.9	(2.6–46.0)
Histology		
Well-differentiated Liposarcoma	24	(43.6%)
Dedifferentiated Liposarcoma	18	(32.7%)
Myxoid liposarcoma	4	(7.3%)
Mixed liposarcoma	7	(12.7%)
Not mentioned	2	(3.6%)

**Fig. 7:**
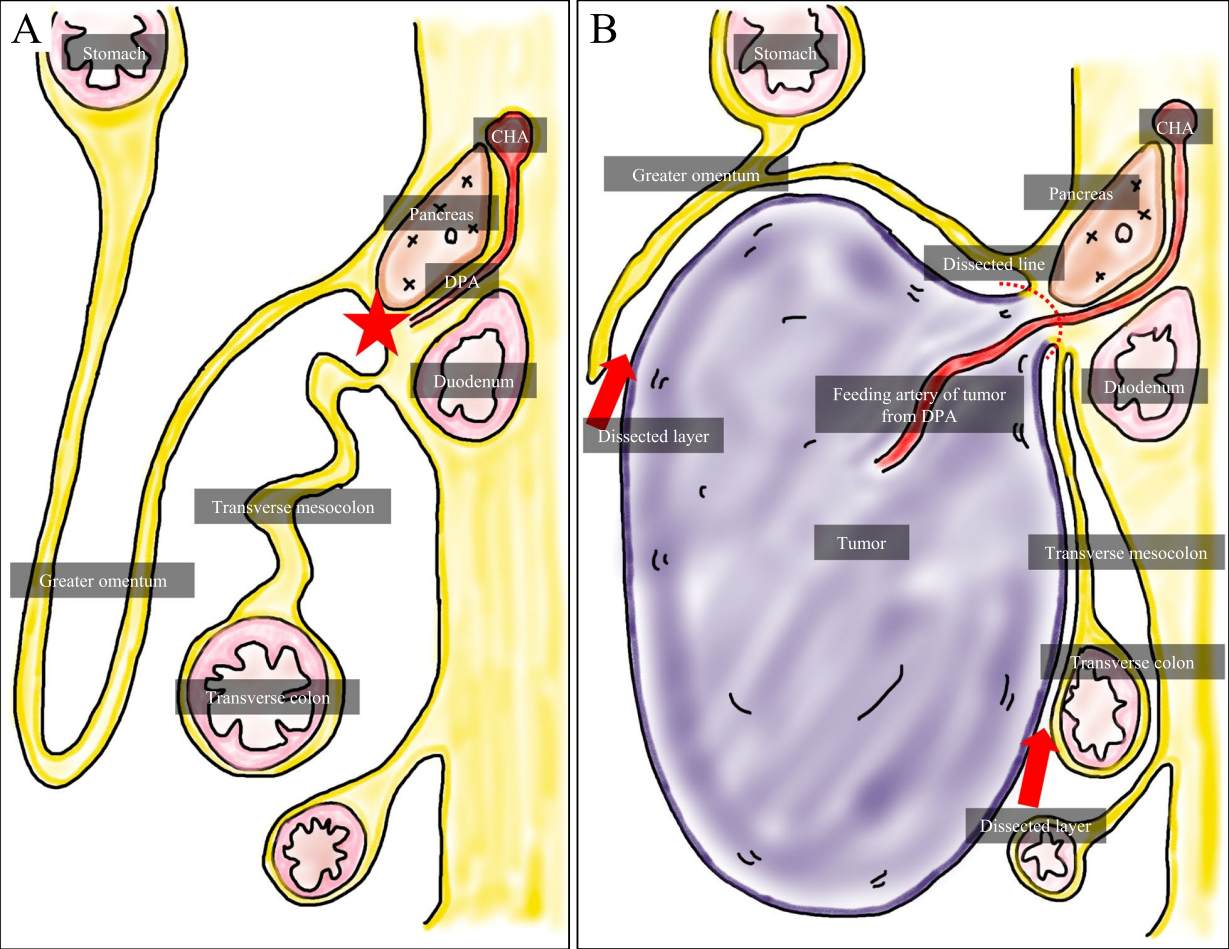
Schema. **A** Embryonic anatomy, **B** Our case. The tumor was supplied by a branch of the DPA, suggesting its origin in the peripancreatic fat tissue (star). Therefore, we considered that the tumor extended from the peripancreatic fat tissue toward the space between the greater omentum and transverse mesocolon. *CHA* common hepatic artery*, DPA* dorsal pancreatic artery

Our case represents a rare origin of retroperitoneum liposarcoma from the peripancreatic fat tissue, and there have been no previous reports of liposarcoma from a similar location. During embryonic development, the greater omentum and transverse mesocolon fuse. We consider that the tumor originated from the peripancreatic fat tissue and extended into the space to abdominal cavity between them, as indicated in the schema (Fig. 7). By utilizing preoperative 3D-CTA, we were able to identify the feeding artery and accurately diagnose the tumor’s origin, leading to a safe and complete resection without injuring the capsule. Without detailed preoperative origin identification, it would have been difficult to determine whether the greater omentum or transverse mesocolon could be dissected, potentially resulting in capsule injury or unnecessary resection of adjacent organs. We were able to treat the patient with a minimally invasive surgery in terms of surgical time, blood loss, and postoperative hospital stay. As mentioned above, among the previously reported cases, feeding artery was identified preoperatively in only three cases. Excluding our case, there are only two cases, one diagnosed with renal artery by angiography in 2010 and another diagnosed with omental artery by contrast-enhanced CT in 2019 [1, 13]. Although angiography is useful for the diagnosis of tumor’s origin, but it is a highly invasive examination and not performed for all patients [60]. In contrast, the 3D-CTA we utilized in our case could be easily created from contrast-enhanced CT and significantly contributed not only to tumor’s origin identification but also to understanding the tumor’s relationship with vessels and facilitating surgical planning. Therefore, we believe that preoperative assessment using 3D-CTA is highly valuable, even for giant tumors, to prevent resorting to a “take potluck” surgery.

## Conclusions

We experienced an extremely rare case of a liposarcoma originating in the peripancreatic fat tissue. We reported the rarity of the tumor’s origin and the utility of preoperative identification and localization of the tumor’s feeding artery using 3D-CTA for achieving complete resection.

## Data Availability

Data sharing is not applicable since no datasets were generated or analyzed during the present study.

## Abbreviations

3D-CTA: 3-Dimensional Computed Tomography Angiography
CT: Computed tomography
MRI: Magnetic response imaging
DPA: Dorsal pancreatic artery
RGEA: Right gastroepiploic artery
SMA: Superior mesenteric artery
MCA: Middle colic artery
MCV: Middle colic vein
RGEV: Right gastroepiploic vein
AcRCV: Accessary right colic vein
